# Association between tongue ultrasonographic characteristics, Yin-deficiency constitution, and intrinsic capacity impairment in older adults: An exploratory cross-sectional study

**DOI:** 10.1097/MD.0000000000049571

**Published:** 2026-07-10

**Authors:** Kuan-Tso Chen, Chia-Ling Chao, Ming-Ta Hsieh, Tsung-Jen Hsieh, Chih-Ting Lin, Po-Huang Chiang, Wen-Long Hu, Chin-Chuan Tsai, I-Cheng Lu, Yu-Chiang Hung

**Affiliations:** a School of Chinese Medicine for Post-Baccalaureate, I-Shou University, Kaohsiung, Taiwan; b Department of Chinese Medicine, E-Da Cancer Hospital, Kaohsiung, Taiwan; c School of Nursing, I-Shou University, Kaohsiung, Taiwan; d Department of Nursing, E-Da Hospital, Kaohsiung, Taiwan; e Department of Family Medicine and Community Medicine, E-Da Hospital, Kaohsiung, Taiwan; f School of Medicine, I-Shou University, Kaohsiung, Taiwan; g Department of Chinese Medicine, Institute of Traditional Medicine, National Yang Ming Chiao Tung University, Taipei, Taiwan; h Institute of Population Health Sciences, National Health Research Institutes, Miaoli, Taiwan; i Global Psychiatric Epidemiology Group, Columbia University Irving Medical Center and New York State Psychiatric Institute, New York, NY; j Department of Chinese Medicine, Kaohsiung Chang Gung Memorial Hospital and Chang Gung University College of Medicine, Kaohsiung, Taiwan; k School of Nursing, FooYin University, Kaohsiung, Taiwan; l School of Medicine, Kaohsiung Medical University, Kaohsiung, Taiwan; m Department of Chinese Medicine, E-Da Dachang Hospital, Kaohsiung, Taiwan; n Department of Chinese Medicine, Linsen Chinese Medicine and Kunming Branch, Taipei City Hospital, Taipei, Taiwan.

**Keywords:** aged, intrinsic capacity, tongue ultrasonography, traditional Chinese medicine constitution, Yin-deficiency

## Abstract

Population aging is accompanied by a progressive decline in intrinsic capacity, yet objective measures reflecting underlying physiological changes remain limited. Yin-deficiency constitution in traditional Chinese medicine has been associated with intrinsic capacity impairment, but its structural correlates remain unclear. This exploratory study investigated associations among tongue ultrasonographic characteristics, Yin-deficiency constitution, and intrinsic capacity impairment in older adults. In this exploratory cross-sectional study, community-dwelling older adults attending a traditional Chinese medicine outpatient clinic (n = 123; mean age 71.6 ± 4.6 years) completed the Constitution in Chinese Medicine Questionnaire–Elderly Edition. Tongue thickness and echo intensity were measured using standardized ultrasonographic protocols, and intrinsic capacity was assessed using the Integrated Care for Older People (ICOPE) framework. Higher tongue echo intensity was associated with lower tongue thickness and higher Yin-deficiency scores. In multivariable linear regression analyses, tongue thickness remained strongly associated with tongue echo intensity (β = −0.548, *P* < .001), whereas the association between Yin-deficiency scores and tongue echo intensity became attenuated and was no longer statistically significant after adjustment for tongue thickness (β = −0.135, *P* = .079). Yin-deficiency constitution was associated with cognitive impairment (odds ratio = 2.79, 95% confidence interval = 1.13–6.89, *P* = .026) and visual impairment (odds ratio = 3.74, 95% confidence interval = 1.57–8.96, *P* = .003), whereas tongue echo intensity alone was not independently associated with ICOPE-defined intrinsic capacity impairments. Thickness-adjusted sensitivity analyses showed attenuated but directionally consistent findings. Tongue echo intensity appears to reflect structural variation in tongue musculature rather than an independent constitution-specific characteristic. Yin-deficiency constitution was associated with cognitive and visual impairment, whereas tongue echo intensity was not independently associated with ICOPE-defined intrinsic capacity impairments. These findings are exploratory and require validation in larger multicenter cohorts.

## 1. Introduction

Population aging is widely recognized as a major global health challenge, as the proportion of older adults continues to increase worldwide, accompanied by a progressive decline in intrinsic capacity and a rising prevalence of chronic diseases. Reliable and accessible approaches to detect early physiological changes are therefore needed to support prevention and intervention strategies in aging populations. Objective measures reflecting biological changes associated with aging have been proposed to help characterize physiological vulnerability and may provide insights into individuals at risk of future functional deterioration.^[1,2]^ Ideally, such measures should be noninvasive, reproducible, and quantitatively assessable, enabling repeated and safe evaluation in both clinical and community settings.^[1]^ Furthermore, integrative approaches may help capture shared physiological alterations that precede clinically apparent disease, highlighting the potential value of objective indicators for early identification of functional decline and intrinsic capacity deterioration.^[2,3]^ Among emerging approaches, imaging-based structural assessment may provide additional insights into age-related tissue characteristics and physiological vulnerability. To operationalize functional vulnerability in older adults, the World Health Organization proposed the Integrated Care for Older People (ICOPE) framework, which prioritizes early detection of declines in intrinsic capacity across multiple functional domains, including cognitive impairment, limited mobility, malnutrition, visual impairment, hearing loss, and depressive symptoms.^[4,5]^ Rather than focusing on disease-specific diagnoses, the ICOPE framework provides a multidimensional and function-oriented approach to assessing age-related vulnerability before overt disability occurs. Recent literature suggests that intrinsic capacity and frailty are complementary but conceptually distinct constructs, with intrinsic capacity emphasizing early detection and prevention at the population level.^[3]^ Therefore, ICOPE provides a clinically relevant framework for examining the relationship between underlying physiological changes and intrinsic capacity impairment in aging populations.

Ultrasonography has been increasingly applied to the assessment of swallowing-related muscles, with parameters such as tongue thickness and echo intensity demonstrating acceptable validity, feasibility, and reproducibility for evaluating swallowing-related function.^[6]^ The tongue is a key effector muscle involved in mastication and swallowing, and age-related alterations in tongue muscle mass and function have been associated with swallowing impairment and sarcopenia-related characteristics in older adults, suggesting that the tongue may provide useful information regarding age-associated functional changes.^[7,8]^ Ultrasonographic measures of the tongue capture complementary aspects of muscle morphology and quality. In particular, echo intensity derived from grayscale imaging may reflect qualitative alterations in tongue muscle, such as increased noncontractile tissue content, and has been associated with reduced tongue pressure and impaired motor performance in older adults.^[9,10]^ However, echo intensity measurements may also be influenced by structural characteristics such as tongue thickness and tissue composition. Previous studies have suggested that tongue echo intensity may be associated with physical frailty and tongue functional performance in older adults, indicating its potential relevance to age-related functional changes.^[11,12]^ Importantly, ultrasound-based tongue assessment is portable, repeatable, and free of radiation, making it suitable for repeated evaluation in clinical and community-based settings.^[6]^ Automated tongue diagnosis systems have been developed to standardize traditional tongue inspection by quantifying surface features such as shape, color, and fur characteristics. However, these approaches are limited to optical and macroscopic observations and may not capture subsurface tissue-level characteristics. Whether constitution-related tongue features correspond to the ultrasonographic characteristics of the underlying tongue muscle structure and quality, therefore, remains unclear.^[13]^

To explore constitution-related characteristics potentially associated with intrinsic capacity impairment, this study adopted the traditional Chinese medicine (TCM) constitution framework, which has increasingly been examined in contemporary research as a clinically meaningful classification rather than a purely theoretical construct.^[14]^ Population-based studies have reported associations between specific TCM constitution types and demographic characteristics, lifestyle factors, physiological traits, and aging-related health conditions, supporting the clinical relevance of constitution-based classification.^[15–17]^ To facilitate standardized constitution assessment, the Constitution in Chinese Medicine Questionnaire (CCMQ), developed by Professor Qi Wang and colleagues, has been widely applied in diverse populations and clinical settings. The CCMQ has demonstrated acceptable reliability and construct validity and has been used to investigate associations between constitution types, chronic diseases, metabolic conditions, and functional health outcomes.^[17,18]^ Given the unique physiological characteristics of older adults, the Constitution in Chinese Medicine Questionnaire–Elderly Edition (CCMQ-EE) has been developed and applied in geriatric populations, showing acceptable reliability and construct validity, and providing a practical tool for constitution-based assessment in aging-related research. Previous studies have reported associations between TCM constitution types assessed using elderly-oriented questionnaires and biomedical indicators as well as age-related diseases, suggesting the potential of constitution measures to complement conventional clinical assessments.^[19,20]^ In our previous cohort study of older adults recruited from community settings and TCM outpatient clinics, Yin-deficiency was the most frequently observed constitution among individuals with ICOPE-defined impairments, with prevalence ranging from 38.5% to 49.3% across abnormal functional domains. Multivariable analyses further showed that Yin-deficiency was associated with cognitive impairment and hearing loss, while both Yin-deficiency and Blood-stasis were associated with visual impairment. In addition, Qi-deficiency and Blood-stasis were associated with malnutrition, and Qi-depression was associated with both visual impairment and hearing loss. These findings suggest that Yin-deficiency may be related to patterns of intrinsic capacity impairment in aging populations.^[21]^ However, these associations should be interpreted with caution, as the underlying mechanisms and causal relationships remain unclear. Despite these advances, the relationship between constitution characteristics and quantitative imaging features remains insufficiently explored. In particular, whether constitution characteristics correspond to tissue-level structural and qualitative features detectable by noninvasive imaging modalities remains unclear. Therefore, combining constitution assessment with objective imaging evaluation may provide additional insights into constitution-related characteristics.

Therefore, this study investigated the associations among tongue ultrasonographic characteristics, Yin-deficiency constitution, and intrinsic capacity impairment in older adults. We further explored the relationships between tongue echo intensity, tongue thickness, and Yin-deficiency constitution, as well as their associations with ICOPE-defined intrinsic capacity impairments. Exploratory analyses were conducted to evaluate the interaction between Yin-deficiency constitution and continuous tongue echo intensity and the potential influence of tongue thickness on these associations.

## 2. Materials and methods

### 2.1. Study population

This study was conducted in accordance with the principles of the Declaration of Helsinki and was approved by the Institutional Review Board of E-Da Hospital (IRB number: EMRP-112-022). Written informed consent was obtained from all participants before enrollment. This exploratory cross-sectional study enrolled older adults aged ≥65 years who attended the outpatient TCM clinics of E-Da Cancer Hospital in southern Taiwan. Participants younger than 65 years were excluded. Recruitment was conducted between May 2023 and October 2025, during which a total of 123 eligible participants were enrolled.

### 2.2. Ultrasonographic measurement of tongue thickness and echo intensity

**Figure 1.**
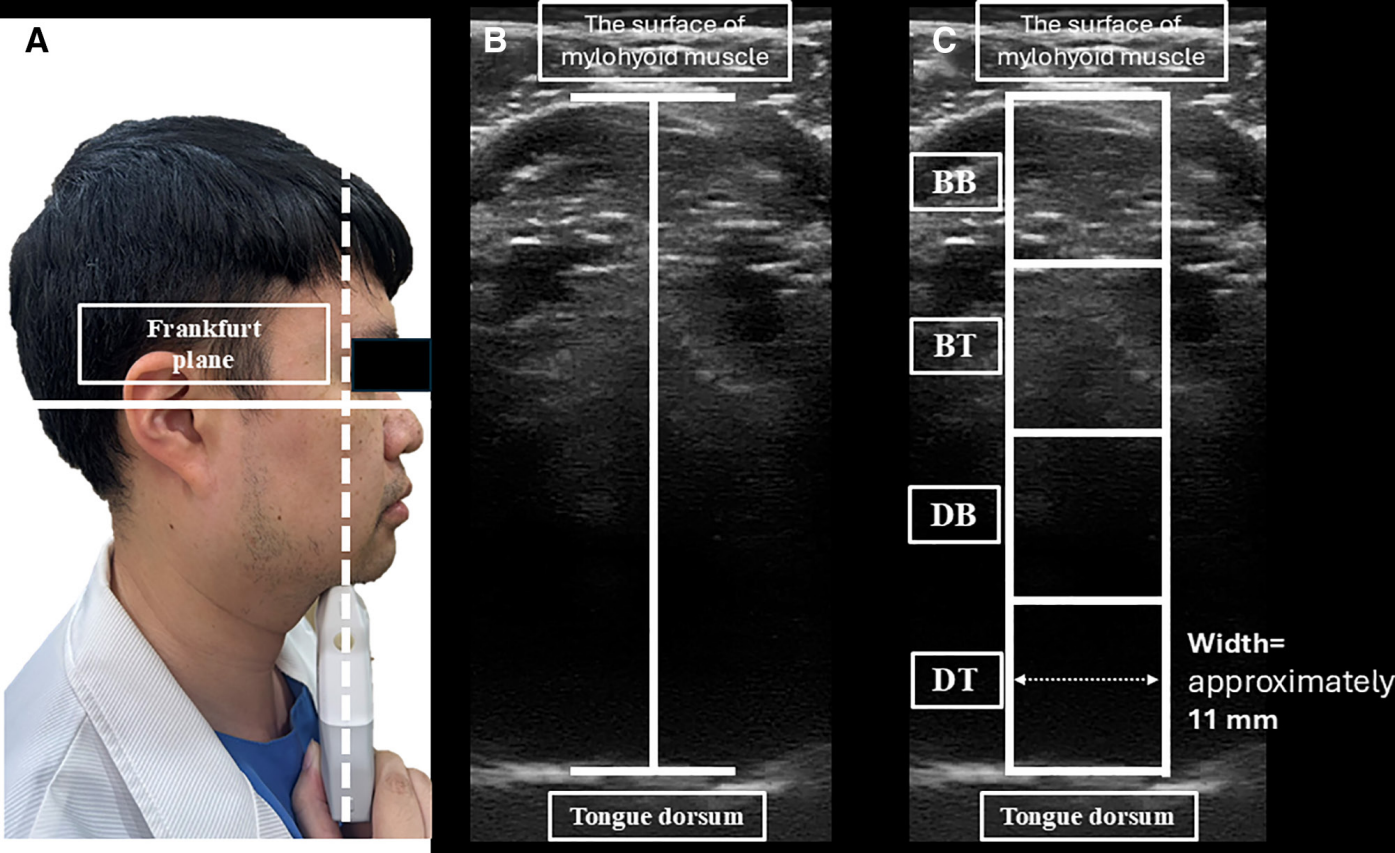
Ultrasonographic assessment of tongue thickness and echo intensity. (A) Participant positioning during tongue ultrasonography, with the head adjusted to maintain the Frankfurt plane parallel to the floor and the probe placed at the midline between the bilateral second premolars. (B) Measurement of tongue thickness on a midsagittal ultrasound image, defined as the vertical distance from the surface of the mylohyoid muscle to the dorsal surface of the tongue. (C) Definition of the ROI for echo intensity analysis, aligned with the axis of tongue thickness measurement. The ROI was vertically subdivided into 4 anatomical regions: top of the tongue dorsal side, bottom of the tongue dorsal side, top of the basal tongue side, and bottom of the basal tongue side. Only the BT region was included in the present analysis. The ROI width was standardized in image units and is shown here with an approximate millimeter scale for reference. BB = basal bottom, BT = basal tongue, DB = dorsal bottom, DT = dorsal tongue, ROI = region of interest.

Tongue ultrasonography was conducted using a handheld ultrasound system (Vscan Air CL, GE Healthcare) with a linear-array probe by a single experienced examiner under standardized conditions. The linear probe was operated using the built-in musculoskeletal preset mode, and all ultrasound examinations were performed using identical acquisition settings. Because of the proprietary design of the handheld ultrasound platform, certain internal image-processing parameters, including dynamic range and post-processing algorithms, were not fully accessible to the investigators. Measurements were obtained exclusively from the top of the basal tongue side (BT), which was selected a priori as the primary region of interest (ROI) for analysis.^[10]^ During the examination, participants were seated upright in a stable chair, with head position adjusted to maintain the Frankfurt plane parallel to the floor and to minimize body movement. The linear-array ultrasound probe (operating frequency range, 3–12 MHz; imaging depth fixed at 8 cm; footprint approximately 40 × 7 mm; mechanical index, 0.8; thermal index, 0.1) was positioned perpendicular to the Frankfurt plane and placed at the midline between the bilateral second premolars (Fig. 1A).

After participants swallowed saliva and assumed the mandibular resting position, a short ultrasound cine loop was recorded once the tongue reached a stable resting state. Tongue thickness was defined as the vertical distance from the surface of the mylohyoid muscle to the dorsal surface of the tongue and was measured on still images extracted from the recorded cine loop (Fig. 1B).

For echo intensity analysis, an ROI was defined along the axis of tongue thickness measurement. The ROI width was standardized to approximately 11 mm across all participants. Although this region can be vertically subdivided into 4 anatomical areas – the top of the tongue dorsal, bottom of the tongue dorsal, BT, and bottom of the basal tongue – only the BT region was included in the present analysis (Fig. 1C).^[10]^

Mean echo intensity values for the BT region were calculated using ImageJ software (National Institutes of Health). All linear measurements, including tongue thickness, were calibrated using a known distance within each ultrasound image, with units expressed in millimeters. For each participant, 3 representative frames with clear and stable visualization of the tongue musculature were selected from the same cine loop, and measurements were performed on each frame. The mean of the 3 frame-based measurements was used as the representative value for subsequent analyses.

Measurement consistency was evaluated using the coefficient of variation calculated from the 3 frame-based measurements within each participant. The mean coefficient of variation values indicated acceptable within-session measurement consistency for both tongue echo intensity (5.96 ± 4.70%) and tongue thickness (0.86 ± 0.89%).

### 2.3. TCM constitution approach

TCM body constitution was assessed using the CCMQ-EE. The CCMQ-EE was developed based on the original CCMQ framework to better accommodate age-related physiological characteristics and symptom expression in older adults, while preserving the original constitution domains and classification principles.

The version used in the present study consisted of 33 items covering 9 TCM constitution types: Balanced, Qi-deficiency, Yang-deficiency, Yin-deficiency, Phlegm-dampness, Damp-heat, Blood-stasis, Qi-depression, and Inherited-special constitution. Items were rated on a 5-point Likert scale, and domain scores were calculated according to the original CCMQ scoring algorithm. A score >17 in the Balanced domain combined with scores ≤8 in all other domains indicated a Balanced constitution, whereas a score ≥11 in any imbalanced domain indicated the corresponding imbalanced constitution.^[22]^ Because multiple constitution types may coexist in older adults, participants could be classified into more than 1 constitution category according to the predefined scoring criteria. In the present sample, the overall internal consistency of the 33-item CCMQ-EE was good (Cronbach α = 0.872). The 33-item CCMQ-EE has been applied in studies of older adults to investigate constitution-related health outcomes.^[23]^

### 2.4. Assessment of intrinsic capacity using the ICOPE framework

Functional impairment was assessed using the World Health Organization ICOPE framework, which encompasses 6 intrinsic capacity domains: cognition, mobility, nutrition, vision, hearing, and psychological well-being. Cognitive function was screened using brief orientation and memory questions addressing temporal orientation, spatial awareness, and short-term recall, aiming to identify early signs of cognitive decline. Mobility was evaluated using the 5-times sit-to-stand test, in which participants were instructed to rise from a seated position 5 consecutive times without using their arms; performance exceeding 12 seconds was considered indicative of mobility limitation. Nutritional status was assessed based on self-reported unintentional weight loss and decreased appetite within the preceding 3 months. Visual function was screened using a near-vision test requiring participants to read standardized text at close distance, while hearing was evaluated using a whisper test or brief hearing screening questions. Psychological well-beingwas assessed using a 2-item screening format addressing persistent sadness and diminished interest or pleasure in usual activities. Each domain was classified as impaired or not impaired according to ICOPE screening criteria, and domain-specific impairments were analyzed individually in exploratory analyses.^[4,5,21]^ The ICOPE screening tool was used to assess intrinsic capacity across multiple domains. It should be noted that ICOPE is designed as a multidomain screening framework for intrinsic capacity rather than a comprehensive frailty assessment.^[3]^

### 2.5. Statistical analysis

All statistical analyses were performed using SPSS version 22.0 (IBM Corp.). Continuous variables are presented as mean ± standard deviation or median (interquartile range), and categorical variables as frequencies and percentages. A two-tailed *P* value <.05 was considered statistically significant. Given the exploratory nature of the study and the multiple comparisons performed, *P* values were interpreted descriptively. Receiver operating characteristic (ROC) analysis was performed exploratorily to evaluate the discriminative performance of tongue echo intensity for Yin-deficiency constitution. The optimal cutoff value was determined using the Youden index and was used only for exploratory categorization analyses. Baseline characteristics between groups were compared using independent *t* tests for continuous variables and chi-square tests for categorical variables. Pearson correlation analyses were conducted to assess the relationships among tongue echo intensity, tongue thickness, and TCM constitution scores. Exploratory logistic regression analyses using the ROC-derived high tongue echo intensity category (>23.53) were performed as supplementary analyses. Variables were first examined using univariate models and subsequently entered into multivariable models to identify factors independently associated with high tongue echo intensity. Odds ratios (ORs) with 95% confidence intervals (CIs) were reported.

To further evaluate structural relationships among Yin-deficiency constitution scores, tongue thickness, and tongue echo intensity, exploratory multivariable linear regression analyses were performed. Standardized beta coefficients (β) were reported to assess the associations between continuous Yin-deficiency scores, tongue thickness, and tongue echo intensity after adjustment for age and sex. Additional thickness-adjusted models were constructed to evaluate attenuation of the association between Yin-deficiency scores and tongue echo intensity. To evaluate functional outcomes, ICOPE-defined impairments were analyzed as binary variables. Exploratory multivariable logistic regression analyses were performed to examine associations between Yin-deficiency constitution, standardized continuous tongue echo intensity (*Z*-score), and domain-specific ICOPE impairments, with adjustment for age and sex. In these models, Yin-deficiency constitution was analyzed as a dichotomous variable based on CCMQ-EE classification, whereas tongue echo intensity was analyzed as a continuous standardized variable. Interaction terms between Yin-deficiency constitution and standardized tongue echo intensity were additionally evaluated. Additional sensitivity analyses were conducted by further adjusting for tongue thickness in interaction models to examine the robustness of the observed associations and the potential influence of tongue structural characteristics. Exploratory cutoff-based combined phenotype analyses using the ROC-derived high tongue echo intensity category together with Yin-deficiency constitution were retained as supplementary analyses. No formal sample size calculation was performed because of the exploratory nature of the study.

## 3. Results

### 3.1. Participant characteristics and grouping by tongue echo intensity

A total of 123 participants were included in the analysis. For descriptive purposes, participants were stratified using the exploratory ROC-derived tongue echo intensity cutoff value of 23.53 into low (≤23.53, n = 46) and high (>23.53, n = 77) echo intensity groups. Compared with the low echo intensity group, participants with high echo intensity showed lower body weight (58.57 ± 8.77 vs 64.26 ± 10.32 kg, *P* = .045), lower tongue thickness (46.56 ± 5.07 vs 51.77 ± 4.82 mm, *P* < .001), and a higher prevalence of Yin-deficiency constitution (36.4% vs 13.0%, *P* = .004; Table 1). Depressive symptoms were more frequently observed in the high echo intensity group in unadjusted comparisons, whereas no statistically significant differences were observed for other ICOPE-defined impairments between groups.

**Table 1:** Exploratory demographic, clinical, and functional characteristics stratified by tongue echo intensity category based on receiver operating characteristic analysis.

Variables	All (n = 123)	Echo intensity ≤ 23.53 (n = 46)	Echo intensity > 23.53 (n = 77)	*P* value
Age (yr)	71.60 ± 4.58	71.72 ± 4.59	71.53 ± 4.60	.841
Sex				
Male	34 (27.6%)	12 (26.1%)	22 (28.6%)	.778
Height (cm)	157.73 ± 7.07	157.10 ± 6.10	158.10 ± 7.61	.436
Weight (kg)	60.70 ± 9.74	64.26 ± 10.32	58.57 ± 8.77	.045*
Tongue thickness (mm)	48.51 ± 5.57	51.77 ± 4.82	46.56 ± 5.07	<.001*
Echo intensity	28.96 ± 13.75	15.29 ± 6.02	37.12 ± 10.08	<.001*
TCM constitution types				
Balanced	41 (33.3%)	17 (37.0%)	24 (31.2%)	.352
Qi-deficiency	19 (15.4%)	4 (8.7%)	15 (19.5%)	.175
Yang-deficiency	21 (17.1%)	6 (13.0%)	15 (19.5%)	.503
Yin-deficiency	34 (27.6%)	6 (13.0%)	28 (36.4%)	.004*
Phlegm-dampness	43 (35.0%)	19 (41.3%)	24 (31.2%)	.345
Damp-heat	8 (6.5%)	2 (4.3%)	6 (7.8%)	.709
Blood-stasis	21 (17.1%)	6 (13.0%)	15 (19.5%)	.503
Qi-depression	22 (17.9%)	9 (19.6%)	13 (16.9%)	.895
Inherited-special	17 (13.8%)	5 (10.9%)	12 (15.6%)	.643
Intrinsic capacity impairment				
Cognitive impairment	44 (35.8%)	17 (37.0%)	27 (35.1%)	.986
Limited mobility	40 (32.5%)	15 (32.6%)	25 (32.5%)	1.000
Malnutrition	11 (8.9%)	3 (6.5%)	8 (10.4%)	.534
Visual impairment	51 (41.5%)	17 (37.0%)	34 (44.2%)	.552
Hearing loss	21 (17.1%)	5 (10.9%)	16 (20.8%)	.244
Depressive symptoms	20 (16.3%)	3 (6.5%)	17 (22.1%)	.044*
No abnormality	26 (21.1%)	10 (21.7%)	16 (20.8%)	1.000
Comorbidities				
Hypertension	48 (39.0%)	22 (47.8%)	26 (33.8%)	.175
Diabetes	24 (19.5%)	11 (23.9%)	13 (16.9%)	.473
Dyslipidemia	28 (22.8%)	13 (28.3%)	15 (19.5%)	.367
Cancer	22 (17.9%)	7 (15.2%)	15 (19.5%)	.723

Continuous variables are presented as mean ± standard deviation and compared using independent *t* tests.

Categorical variables are presented as n (%) and compared using chi-square tests.

TCM = traditional Chinese medicine.

* *P* < .05.

### 3.2. Correlations between tongue echo intensity and clinical variables

Tongue echo intensity was strongly negatively correlated with tongue thickness (*r* = −0.578, *P* < .001) and showed weaker correlations with body weight (*r* = −0.262, *P* = .004) and Yin-deficiency constitution scores (*r* = 0.202, *P* = .025; Table S1, Supplemental Digital Content 1). Additional weak correlations with other constitution scores are presented in Table S1, Supplemental Digital Content 1.

### 3.3. ROC analysis of tongue echo intensity for distinguishing Yin-deficiency constitution

**Figure 2.**
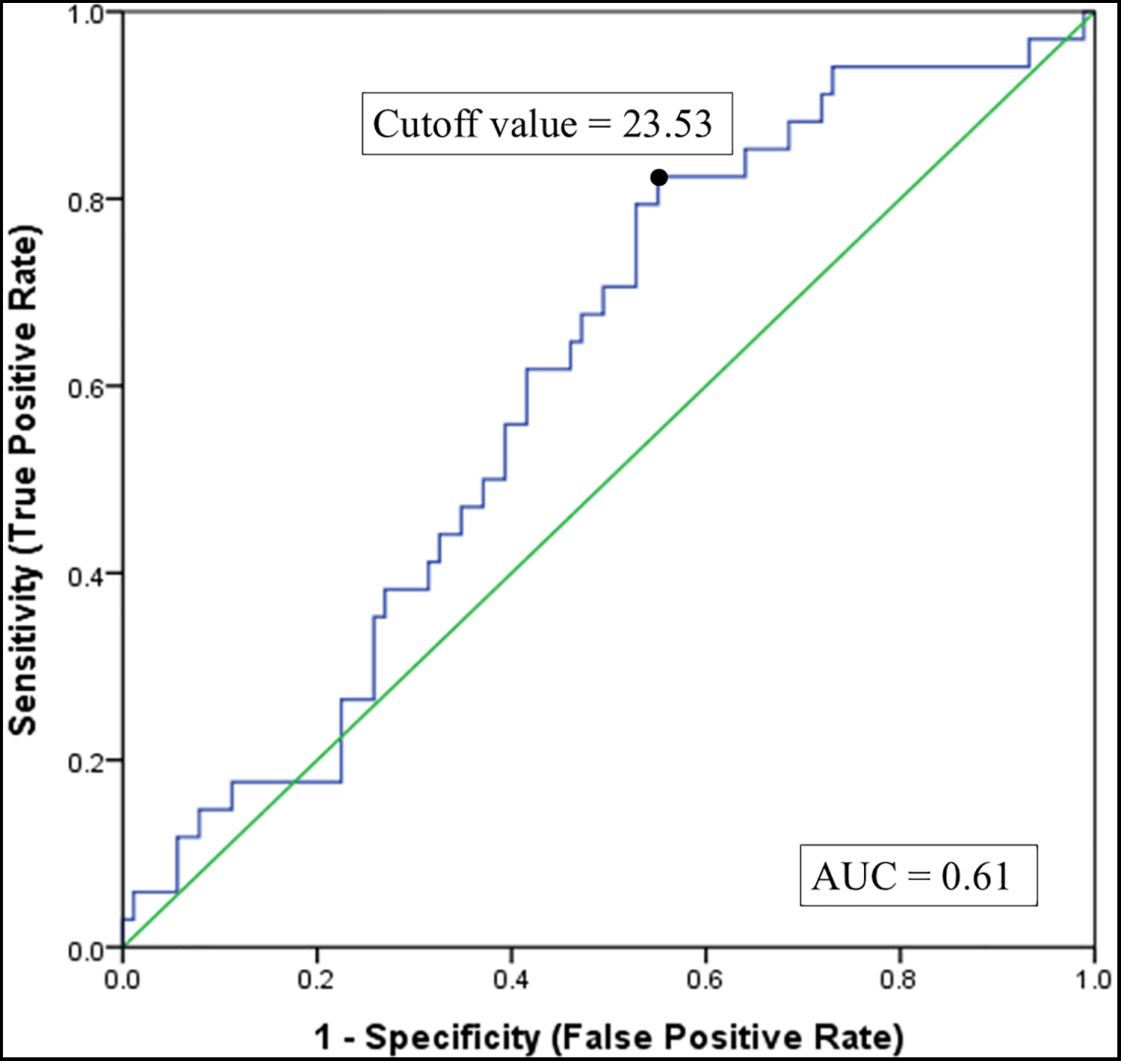
Exploratory ROC analysis of tongue echo intensity for distinguishing Yin-deficiency constitution. The area under the ROC curve (AUC) was 0.61, indicating limited discriminative performance and suggesting that echo intensity alone may not serve as a standalone classifier. The optimal cutoff value determined by the Youden index was 23.53, yielding a sensitivity of 82.4% and a specificity of 44.9%. AUC = area under the curve, ROC = receiver operating characteristic.

ROC analysis showed limited discriminative performance of tongue echo intensity for distinguishing Yin-deficiency constitution (area under the curve [AUC] = 0.61; Fig. 2). The optimal cutoff value determined by the Youden index was 23.53, with a sensitivity of 82.4% and a specificity of 44.9%. This cutoff was retained only for supplementary categorization analyses.

### 3.4. Structural associations among tongue echo intensity, tongue thickness, and Yin-deficiency constitution

Univariate logistic regression analyses showed that lower body weight, reduced tongue thickness, and Yin-deficiency constitution were associated with high tongue echo intensity (Table 2). In the multivariable model, tongue thickness remained significantly associated with high echo intensity (OR = 0.84, 95% CI = 0.77–0.93, *P* < .001), whereas the association between Yin-deficiency constitution and high tongue echo intensity became attenuated and was no longer statistically significant after adjustment for tongue thickness (OR = 2.36, 95% CI = 0.83–6.70, *P* = .107).

**Table 2:** Exploratory logistic regression analyses of factors associated with high tongue echo intensity based on receiver operating characteristic analysis.

Variables	OR (95% CI)	*P* value
Univariate analysis		
Age	0.99 (0.92–1.07)	.828
Male	1.13 (0.50–2.58)	.766
Height	1.02 (0.97–1.08)	.449
Weight	0.94 (0.90–0.98)	.003*
Tongue thickness (mm)	0.81 (0.74–0.89)	<.001*
Balanced	0.70 (0.33–1.51)	.368
Qi-deficiency	2.54 (0.79–8.19)	.118
Yang-deficiency	1.61 (0.58–4.50)	.362
Yin-deficiency	3.81 (1.44–10.11)	.007*
Phlegm-dampness	0.64 (0.30–1.38)	.255
Damp-heat	1.86 (0.36–9.62)	.460
Blood-stasis	1.61 (0.58–4.50)	.362
Qi-depression	0.84 (0.33–2.14)	.707
Inherited-special	1.51 (0.50–4.61)	.466
Hypertension	0.56 (0.26–1.17)	.124
Diabetes	0.65 (0.26–1.59)	.343
Dyslipidemia	0.61 (0.26–1.44)	.263
Cancer	1.35 (0.50–3.60)	.551
Multivariate analysis		
Weight	0.97 (0.93–1.01)	.183
Tongue thickness (mm)	0.84 (0.77–0.93)	<.001*
Yin-deficiency	2.36 (0.83–6.70)	.107

High tongue echo intensity was defined using the ROC-derived cutoff value of >23.53.

CI = confidence interval, OR = odds ratio, ROC = receiver operating characteristic.

* *P* < .05.

Multivariable linear regression analyses further showed that higher Yin-deficiency scores were associated with higher tongue echo intensity (β = 0.230, *P* = .012) and lower tongue thickness (β = −0.261, *P* = .004) after adjustment for age and sex (Table 3). In thickness-adjusted models, tongue thickness remained strongly associated with tongue echo intensity (β = −0.548, *P* < .001), whereas the positive association between Yin-deficiency scores and tongue echo intensity became attenuated and was no longer statistically significant after adjustment for tongue thickness (β = −0.135, *P* = .079).

**Table 3:** Exploratory regression analyses of associations among Yin-deficiency scores, tongue thickness, and tongue echo intensity.

Dependent variable	Predictor	β	*P* value
Tongue echo intensity	Yin-deficiency score	0.230	.012*
	Age	0.163	.072
	Male sex	0.039	.662
Tongue thickness	Yin-deficiency score	−0.261	.004*
	Age	−0.142	.111
	Male sex	0.113	.207
Tongue echo intensity (thickness-adjusted model)	Tongue thickness	−0.548	<.001*
	Yin-deficiency score	−0.135	.079
	Age	−0.053	.482
	Male sex	0.134	.073

Models were adjusted for age and sex.

“β” represents standardized regression coefficients.

The thickness-adjusted model additionally included tongue thickness as a covariate.

* *P* < .05.

### 3.5. Sensitivity analyses

Sensitivity analyses using multiple logistic regression models showed that Yin-deficiency constitution was associated with high tongue echo intensity in unadjusted analyses (OR = 3.81, 95% CI = 1.44–10.11, *P* = .007; Table S2, Supplemental Digital Content 2). Tongue thickness consistently demonstrated a strong inverse association with high tongue echo intensity across models (OR range = 0.81–0.84, all *P* < .001). After adjustment for tongue thickness, the association between Yin-deficiency and high tongue echo intensity became attenuated and was no longer statistically significant (OR = 2.41, 95% CI = 0.85–6.80, *P* = .097). These findings further supported the robustness of the inverse association between tongue thickness and tongue echo intensity and suggested that tongue thickness may contribute to the observed association between Yin-deficiency constitution and tongue echo intensity.

### 3.6. Associations between Yin-deficiency constitution, continuous tongue echo intensity, and cognitive impairment

Multivariable logistic regression analyses showed that Yin-deficiency constitution was associated with cognitive impairment after adjustment for age and sex (OR = 2.79, 95% CI = 1.13–6.89, *P* = .026), whereas standardized continuous tongue echo intensity alone was not significantly associated with cognitive impairment (Table 4). No statistically significant interaction between Yin-deficiency constitution and standardized tongue echo intensity was observed (OR = 2.00, 95% CI = 0.80–4.99, *P* = .138). In thickness-adjusted analyses, the association between Yin-deficiency constitution and cognitive impairment became attenuated (OR = 2.45, 95% CI = 0.97–6.16, *P* = .057; Table 5).

**Table 4:** Multivariable logistic regression analysis of cognitive impairment including the interaction between Yin-deficiency constitution and continuous tongue echo intensity.

Variable	OR (95% CI)	*P* value
Age	1.13 (1.04–1.24)	.006*
Male	1.21 (0.50–2.95)	.676
Yin-deficiency	2.79 (1.13–6.89)	.026*
Standardized tongue echo intensity (*Z*-score)	0.73 (0.44–1.19)	.202
Yin-deficiency × echo intensity (*Z*-score) interaction	2.00 (0.80–4.99)	.138

Data are presented as ORs with 95% CIs.

The model was adjusted for age and sex.

Standardized tongue echo intensity was analyzed as a continuous variable using *Z*-score transformation.

Yin-deficiency constitution was analyzed as a dichotomous variable based on CCMQ-EE classification.

The interaction term was defined as Yin-deficiency constitution × standardized tongue echo intensity.

CCMQ-EE = Constitution in Chinese Medicine Questionnaire–Elderly Edition, CI = confidence interval, OR = odds ratio.

* *P* < .05.

**Table 5:** Thickness-adjusted multivariable logistic regression analysis of cognitive impairment including the interaction between Yin-deficiency constitution and continuous tongue echo intensity.

Variable	OR (95% CI)	*P* value
Age	1.13 (1.03–1.24)	.008*
Male	1.34 (0.54–3.35)	.529
Yin-deficiency	2.45 (0.97–6.16)	.057
Standardized tongue echo intensity (*Z*-score)	0.57 (0.32–1.04)	.069
Yin-deficiency × echo intensity (*Z*-score) interaction	2.20 (0.87–5.59)	.097
Tongue thickness	0.93 (0.85–1.03)	.158

Data are presented as ORs with 95% CIs.

The model was adjusted for age and sex.

Standardized tongue echo intensity was analyzed as a continuous variable using *Z*-score transformation.

Yin-deficiency constitution was analyzed as a dichotomous variable based on CCMQ-EE classification.

The interaction term was defined as Yin-deficiency constitution × standardized tongue echo intensity.

CCMQ-EE = Constitution in Chinese Medicine Questionnaire–Elderly Edition, CI = confidence interval, OR = odds ratio.

* *P* < .05.

### 3.7. Associations between Yin-deficiency constitution, continuous tongue echo intensity, and visual impairment

Multivariable logistic regression analyses showed that Yin-deficiency constitution was associated with visual impairment after adjustment for age and sex (OR = 3.74, 95% CI = 1.57–8.96, *P* = .003), whereas standardized continuous tongue echo intensity alone was not significantly associated with visual impairment (Table 6). No statistically significant interaction between Yin-deficiency constitution and tongue echo intensity was observed. In thickness-adjusted analyses, the association between Yin-deficiency constitution and visual impairment remained statistically significant and directionally consistent (OR = 3.53, 95% CI = 1.45–8.57, *P* = .005; Table 7).

**Table 6:** Multivariable logistic regression analysis of visual impairment including the interaction between Yin-deficiency constitution and continuous tongue echo intensity.

Variable	OR (95% CI)	*P* value
Age	1.04 (0.95–1.13)	.392
Male	0.83 (0.35–1.99)	.682
Yin-deficiency	3.74 (1.57–8.96)	.003*
Standardized tongue echo intensity (*Z*-score)	0.89 (0.56–1.41)	.613
Yin-deficiency × echo intensity (*Z*-score) interaction	1.36 (0.56–3.32)	.502

Data are presented as ORs with 95% CIs.

The model was adjusted for age and sex.

Standardized tongue echo intensity was analyzed as a continuous variable using *Z*-score transformation.

Yin-deficiency constitution was analyzed as a dichotomous variable based on CCMQ-EE classification.

The interaction term was defined as Yin-deficiency constitution × standardized tongue echo intensity.

CCMQ-EE = Constitution in Chinese Medicine Questionnaire–Elderly Edition, CI = confidence interval, OR = odds ratio.

* *P* < .05.

**Table 7:** Thickness-adjusted multivariable logistic regression analysis of visual impairment, including the interaction between Yin-deficiency constitution and continuous tongue echo intensity.

Variable	OR (95% CI)	*P* value
Age	1.04 (0.95–1.13)	.413
Male	0.87 (0.36–2.10)	.758
Yin-deficiency	3.53 (1.45–8.57)	.005*
Standardized tongue echo intensity (*Z*-score)	0.80 (0.46–1.39)	.429
Yin-deficiency × echo intensity (*Z*-score) interaction	1.42 (0.57–3.50)	.452
Tongue thickness	0.97 (0.88–1.06)	.501

Data are presented as ORs with 95% CIs.

The model was adjusted for age and sex.

Standardized tongue echo intensity was analyzed as a continuous variable using *Z*-score transformation.

Yin-deficiency constitution was analyzed as a dichotomous variable based on CCMQ-EE classification.

The interaction term was defined as Yin-deficiency constitution × standardized tongue echo intensity.

CCMQ-EE = Constitution in Chinese Medicine Questionnaire–Elderly Edition, CI = confidence interval, OR = odds ratio.

* *P* < .05.

### 3.8. Supplementary exploratory analyses using ROC-derived tongue echo intensity categories

Supplementary analyses using the ROC-derived high tongue echo intensity category (>23.53) are presented in Tables S3 and S4, Supplemental Digital Content 3 and 4. Participants with both high tongue echo intensity and Yin-deficiency constitution showed higher odds of cognitive impairment (OR = 2.54, 95% CI = 1.04–6.22, *P* = .041) and visual impairment (OR = 3.38, 95% CI = 1.39–8.24, *P* = .007) in age- and sex-adjusted exploratory models. No significant associations were observed for other ICOPE-defined impairments. These findings should be interpreted cautiously because the analyses were exploratory, based on ROC-derived categorization, and not externally validated.

## 4. Discussion

### 4.1. Principal findings

To our knowledge, this study is among the first to integrate tongue ultrasonography with constitution-based assessment within the World Health Organization ICOPE framework. By combining tongue ultrasonographic characteristics with constitution assessment, this exploratory study examined potential relationships among tongue structure, Yin-deficiency constitution, and intrinsic capacity impairment in older adults.

Several observations from the present study warrant discussion. First, tongue echo intensity showed a consistent inverse association with tongue thickness, suggesting that tongue echo intensity is influenced by structural characteristics of tongue musculature. The consistency of this association across multiple analyses indicates that tongue thickness may be an important structural correlate of tongue echo intensity in older adults.

Second, tongue echo intensity demonstrated limited discriminative performance for distinguishing Yin-deficiency constitution (AUC = 0.61). Although tongue echo intensity was positively associated with Yin-deficiency constitution in unadjusted analyses, this association became attenuated and was no longer statistically significant after adjustment for tongue thickness. These findings suggest that tongue thickness may partially account for the observed relationship between Yin-deficiency constitution and tongue echo intensity, and that tongue echo intensity should not currently be interpreted as an independent constitution-specific biomarker.

Third, Yin-deficiency constitution was associated with cognitive and visual impairment, whereas tongue echo intensity alone was not independently associated with ICOPE-defined intrinsic capacity impairments. Exploratory analyses using ROC-derived tongue echo intensity categories suggested possible associations with cognitive and visual impairment; however, these findings should be interpreted cautiously because the analyses were exploratory, based on ROC-derived categorization, and not externally validated. Constitution assessment alone demonstrated similar or stronger associations with intrinsic capacity impairment, suggesting that tongue ultrasonography did not demonstrate clear incremental predictive value beyond constitution assessment in the present study. These findings should therefore be regarded as hypothesis-generating and require validation in larger independent cohorts.

### 4.2. Ultrasonographic correlates of tongue muscle characteristics

Although age-related muscle weakness and atrophy are widely recognized, age was not independently associated with tongue echo intensity in the present study. This observation is consistent with previous studies reporting no significant correlation between chronological age and tongue muscle volume.^[9,24]^ These findings suggest that variation in tongue echo characteristics may not be determined solely by aging itself but may also reflect localized structural alterations and other biological factors.

Previous studies have shown that tongue echo intensity is associated with tongue thickness, tongue pressure, motor performance, and frailty-related characteristics in older adults.^[6,9,11]^ In addition, ultrasonographic assessment of tongue and swallowing-related muscles has been applied in the evaluation of swallowing impairment and dysphagia.^[6]^ Echo intensity derived from grayscale ultrasonography has been proposed as an indicator of muscle quality, reflecting qualitative changes in muscle tissue beyond simple muscle quantity. Increased echo intensity has been associated with aging-related alterations in muscle composition, including increased intramuscular adipose tissue and other noncontractile components.^[25]^ Consistent with these observations, tongue thickness was the most robust structural correlate of tongue echo intensity across multivariable and sensitivity analyses in the present study. Previous studies have similarly identified tongue thickness at both the middle and basal regions of the tongue as significant determinants of tongue echo intensity.^[9]^ The inverse association between tongue thickness and tongue echo intensity observed in our cohort therefore supports the interpretation that echo intensity is strongly influenced by underlying tongue muscle structure.

Additional evidence from studies of sarcopenic dysphagia further supports the relevance of tongue muscle morphology in age-related swallowing dysfunction. Tongue muscle area has been reported to be associated with sarcopenic dysphagia and may reflect advanced structural changes of tongue musculature.^[26]^ Although our study did not evaluate dysphagia directly, these findings provide biological context for interpreting tongue ultrasonographic characteristics in older adults. In the present study, echo intensity was measured in the BT region, which primarily contains extrinsic tongue muscles, including the genioglossus and hyoglossus. Previous studies have demonstrated that echo intensity in the BT region is inversely associated with tongue thickness and tongue pressure, suggesting that this region may provide a relatively stable and functionally relevant site for assessing tongue muscle characteristics in older adults.^[10]^ However, differences in ultrasound systems, acquisition settings, and image-processing algorithms should be considered when comparing absolute echo intensity values across studies. Importantly, tongue echo intensity demonstrated only limited discriminative performance for distinguishing Yin-deficiency constitution (AUC = 0.61). Together with the attenuation of associations after adjustment for tongue thickness, these findings suggest that tongue echo intensity primarily reflects structural variation in tongue musculature and should not currently be interpreted as an independent constitution-specific biomarker.

### 4.3. Constitution characteristics and biological interpretation

TCM clinical assessment is classically based on 4 diagnostic modalities: observation, auscultation and olfaction, inquiry, and palpation.^[27]^ Within this framework, tongue examination represents a key observational method and includes evaluation of tongue color, coating characteristics, surface morphology, and sublingual vascular patterns. Beyond its role in evaluating current conditions, tongue examination has also been considered to reflect constitution-related characteristics.

In recent years, TCM constitution has increasingly been examined as a relatively stable clinical classification associated with long-term physiological tendencies rather than transient symptoms. Previous epidemiological studies have reported associations between constitution types and aging-related functional characteristics, including frailty-related features, in older adults.^[22]^ Among these constitution types, Yin-deficiency has been associated with intrinsic capacity impairment and aging-related symptoms in older adults.^[21]^

Within TCM theory, Yin-deficiency is traditionally described as a state characterized by relative insufficiency of body fluids and reduced tissue nourishment.^[28]^ From a biomedical perspective, these concepts may be broadly related to aging-associated physiological changes, including alterations in tissue composition and skeletal muscle structure.^[25,29]^ However, tissue-level structural correlates of constitution characteristics remain insufficiently explored.

Consistent with this perspective, correlation analyses in the present study demonstrated that tongue echo intensity was inversely associated with tongue thickness and weakly associated with several constitution scores, including Yin-deficiency. Importantly, the association between Yin-deficiency and tongue echo intensity became attenuated after adjustment for tongue thickness, suggesting that tongue structural characteristics may partially contribute to this relationship. These findings support the interpretation that tongue echo intensity may reflect underlying structural variation in tongue musculature rather than simple imaging variability alone.

Within the exploratory framework of the present study, tongue ultrasonography may provide objective structural information relevant to constitution-related characteristics in older adults.

However, tongue echo intensity should not currently be interpreted as an independent biomarker of constitution status.

Exploratory interaction analyses further showed associations between Yin-deficiency constitution and cognitive and visual impairment, whereas tongue echo intensity alone was not independently associated with ICOPE-defined intrinsic capacity impairments. These observations require cautious interpretation and further validation in larger independent cohorts.

### 4.4. Integrative aging framework and clinical implications

The integration of constitution-based assessment with objective imaging modalities represents a potential direction for integrative aging research. Conventional tongue diagnosis, including automated tongue diagnosis systems, has primarily focused on surface-level optical features.^[13]^ In contrast, ultrasonographic assessment enables visualization of subsurface tongue structure and quantitative evaluation of tongue muscle characteristics. The present findings suggest that constitution assessment and tongue ultrasonography may provide complementary structural information regarding constitution-related characteristics and structural variation in older adults. From an integrative aging perspective, functional decline in later life is increasingly recognized as a multidimensional process involving interactions among biological, structural, and functional domains.^[30]^ Previous studies have reported associations between TCM constitution and frailty-related characteristics in older adults,^[22]^ while ultrasonography-derived echo intensity has been associated with muscle quality and frailty-related features.^[31]^ Constitution types have also been linked to multiple geriatric functional domains, including cognition and activities of daily living.^[21,32]^ Consistent with these observations, our previous community-based study demonstrated that older adults with ICOPE-defined intrinsic capacity impairment showed a higher prevalence of Yin-deficiency constitution.^[21]^ In the present study, tongue thickness was the strongest structural correlate of tongue echo intensity, whereas Yin-deficiency constitution, rather than tongue echo intensity alone, was associated with selected intrinsic capacity impairments. These findings suggest that constitution characteristics, tongue structure, and imaging-derived features may represent related aspects of aging-associated physiological variation rather than independent markers. Exploratory analyses further suggested that tongue thickness may partially account for the observed association between Yin-deficiency constitution and tongue echo intensity. However, causal mediation cannot be established because of the cross-sectional study design. Therefore, these findings should be interpreted cautiously, and longitudinal studies are needed to clarify the temporal relationships among constitution characteristics, tongue structure, and intrinsic capacity impairment. Importantly, tongue ultrasonography did not demonstrate clear independent incremental predictive value beyond Yin-deficiency constitution in the present exploratory analyses. Nevertheless, tongue ultrasonography may provide objective structural information regarding tongue musculature that could complement constitution assessment in future research settings. At present, the potential role of tongue ultrasonography within integrative aging assessment frameworks remains exploratory and requires prospective validation before clinical implementation. Although tongue ultrasonography did not demonstrate clear independent associations with ICOPE-defined intrinsic capacity impairments, it may still provide complementary structural information during integrative geriatric assessment. For example, among older adults with Yin-deficiency constitution who also present with swallowing-related symptoms or nutritional concerns, ultrasonographic evaluation may help characterize underlying tongue muscle structure and identify features potentially related to muscle quality or age-related functional vulnerability. Such information may contribute to individualized assessment and provide a framework for future studies investigating constitution-based interventions and tongue muscle-targeted strategies in aging populations. The absence of an independent association between tongue echo intensity and ICOPE-defined impairment should also be interpreted in the context of the assessment framework used in this study. ICOPE was developed as a population-level screening framework for intrinsic capacity rather than a comprehensive frailty assessment instrument. Recent literature suggests that intrinsic capacity and frailty are complementary but conceptually distinct constructs.^[3]^ Therefore, structural variations detected by tongue ultrasonography may not necessarily be reflected in ICOPE-defined impairments, particularly among relatively well-functioning older adults.

Experimental studies have suggested that traditional herbal formulas used for Yin-deficiency-related conditions, such as Jaeumganghwa-Tang, may influence muscle-related pathways in aging models. Although the clinical relevance of these findings to tongue structural characteristics and intrinsic capacity impairment remains unclear, they may provide a rationale for future mechanistic and translational studies investigating constitution characteristics, tongue ultrasonographic features, and aging-related functional outcomes.^[33]^

### 4.5. Limitations

Several limitations should be acknowledged. First, the cross-sectional design precludes causal inference regarding the relationships among constitution characteristics, tongue structure, tongue echo intensity, and intrinsic capacity impairment. Although exploratory analyses suggested that tongue thickness may partially account for the association between Yin-deficiency constitution and tongue echo intensity, causal mediation could not be established. Future longitudinal studies are needed to clarify the temporal and biological relationships among these factors.

Second, participants were recruited from a single TCM outpatient clinic, which may limit generalizability and introduce potential selection bias. Several potentially important confounders were not available in the present study, including comprehensive frailty assessments, sarcopenia-related measures, detailed nutritional evaluation, oral health status, educational level, physical activity, socioeconomic characteristics, systemic inflammation, and ongoing herbal treatment. Although selected functional and nutritional domains were partially captured through the ICOPE framework, ICOPE is a screening tool for intrinsic capacity and does not replace detailed frailty, sarcopenia, or nutritional assessments. Therefore, residual confounding cannot be excluded.

Third, ultrasonographic measurements were performed by a single examiner using a standardized protocol, and inter-rater reliability was not assessed. Echo intensity measurements may be influenced by device-specific settings, image-processing algorithms, and hardware variability across ultrasound systems. In addition, dynamic range settings and proprietary post-processing parameters were not fully accessible on the handheld ultrasound system used in this study, and no tissue-mimicking phantom calibration was performed. Therefore, absolute echo intensity values should be interpreted cautiously and may not be directly comparable across different ultrasound platforms. Finally, multiple exploratory analyses were conducted without formal adjustment for multiple comparisons, increasing the potential risk of type I error. In addition, tongue-specific functional assessments, such as tongue pressure and oral diadochokinesis, were not included. Therefore, statistically significant findings should be interpreted cautiously and regarded as hypothesis-generating. Future multicenter studies with larger sample sizes, standardized ultrasonographic protocols, external validation cohorts, longitudinal follow-up, and comprehensive geriatric assessments are needed to confirm the reproducibility and clinical relevance of these findings.

## 5. Conclusion

In conclusion, tongue echo intensity assessed by ultrasonography was inversely associated with tongue thickness and showed a modest association with Yin-deficiency constitution in older adults. After adjustment for tongue thickness, the association between Yin-deficiency constitution and tongue echo intensity became attenuated, suggesting that tongue structural characteristics may partially account for this relationship. These findings indicate that tongue echo intensity primarily reflects structural variation in tongue musculature rather than an independent constitution-specific characteristic. Yin-deficiency constitution was associated with cognitive and visual impairment, whereas tongue echo intensity alone was not independently associated with ICOPE-defined intrinsic capacity impairments. In addition, tongue echo intensity demonstrated limited discriminative performance for distinguishing Yin-deficiency constitution (AUC = 0.61), suggesting limited utility as a standalone constitution biomarker. Given the exploratory nature of the study, the cross-sectional design, and the multiple comparisons performed, these findings should be interpreted cautiously and regarded as hypothesis-generating. Further longitudinal, multicenter, and externally validated studies are needed to clarify the biological and clinical relevance of tongue ultrasonographic characteristics in aging populations.

## Acknowledgments

The authors sincerely acknowledge Professor Qi Wang for his pioneering and foundational work in traditional Chinese medicine constitution theory, which underpins this study, and for authorizing the use of the Constitution in Chinese Medicine Questionnaire–Elderly Edition (CCMQ-EE). We also thank all participants for their valuable contributions.

## Author contributions

**Conceptualization:** Kuan-Tso Chen, Chia-Ling Chao, Yu-Chiang Hung.

**Data curation:** Kuan-Tso Chen, Chia-Ling Chao, Po-Huang Chiang.

**Formal analysis:** Kuan-Tso Chen, Tsung-Jen Hsieh, I-Cheng Lu.

**Methodology:** Kuan-Tso Chen, Chia-Ling Chao, Ming-Ta Hsieh, Tsung-Jen Hsieh, I-Cheng Lu.

**Visualization:** Kuan-Tso Chen.

**Investigation:** Chia-Ling Chao, Chin-Chuan Tsai.

**Software:** Chih-Ting Lin.

**Resources:** Po-Huang Chiang.

**Validation:** Po-Huang Chiang, I-Cheng Lu.

**Project administration:** Wen-Long Hu.

**Supervision:** Wen-Long Hu, Yu-Chiang Hung.

**Funding acquisition:** Yu-Chiang Hung.

**Writing – original draft:** Kuan-Tso Chen.

**Writing – review & editing:** Chia-Ling Chao, Chih-Ting Lin, I-Cheng Lu, Yu-Chiang Hung.








