# Supplementary material for: Association between tongue ultrasonographic characteristics, Yin-deficiency constitution, and intrinsic capacity impairment in older adults: An exploratory cross-sectional study
Source: Medicine (Baltimore). 2026 Jul 10;105(28):e49571. doi: 10.1097/MD.0000000000049571 (PMC13363339; doi:10.1097/MD.0000000000049571)
Supplement: Supplementary file 1 [file medi-105-e49571-s001.docx]

**Table S1. Exploratory correlations between tongue echo intensity, tongue thickness, clinical variables, and traditional Chinese medicine constitution scores**

| **Variables** | **r** | **95% CI** | ***P*-value** |
| --- | --- | --- | --- |
| Tongue thickness | -0.578 | -0.684 – -0.449 | < .001* |
| Weight | -0.262 | -0.390 – -0.137 | .004* |
| Yin-deficiency | 0.202 | 0.039 – 0.366 | .025* |
| Damp-heat | 0.209 | 0.037 – 0.384 | .020* |
| Yang-deficiency | 0.193 | 0.050 – 0.328 | .032* |
| Qi-deficiency | 0.157 | -0.008 – 0.312 | .083 |
| Phlegm-dampness | -0.062 | -0.225 – 0.098 | .495 |
| Blood-stasis | 0.176 | -0.023 – 0.321 | .052 |
| Qi-depression | 0.092 | -0.057 – 0.250 | .313 |
| Inherited special | 0.258 | 0.098 – 0.416 | .004* |

Values are presented as Pearson correlation coefficients (r) with 95% confidence intervals (CI).

An asterisk (*) indicates *P* < 0.05.
