# Supplementary material for: Association between tongue ultrasonographic characteristics, Yin-deficiency constitution, and intrinsic capacity impairment in older adults: An exploratory cross-sectional study
Source: Medicine (Baltimore). 2026 Jul 10;105(28):e49571. doi: 10.1097/MD.0000000000049571 (PMC13363339; doi:10.1097/MD.0000000000049571)
Supplement: Supplementary file 2 [file medi-105-e49571-s002.docx]

**Table S2. Exploratory stepwise sensitivity analyses of logistic regression models using the tongue echo intensity cutoff derived from receiver operating characteristic analysis**

| **Model** | **Variable** | **OR (95% CI)** | ***P*-value** |
| --- | --- | --- | --- |
| Model 1 | Yin-deficiency | 3.81 (1.44–10.11) | .007* |
| Model 2 | Tongue thickness | 0.81 (0.74–0.89) | < .001* |
| Model 3 | Weight | 0.94 (0.90–0.98) | .003* |
| Model 4 | Yin-deficiency | 2.41 (0.85–6.80) | .097 |
|  | Tongue thickness | 0.83 (0.76–0.90) | < .001* |
| Model 5 | Yin-deficiency | 3.50 (1.29–9.49) | .014* |
|  | Weight | 0.94 (0.90–0.98) | .006* |
| Model 6 | Weight | 0.97 (0.93–1.01) | .164 |
|  | Tongue thickness | 0.83 (0.76–0.91) | < .001* |
| Model 7 (Full) | Yin-deficiency | 2.36 (0.83–6.70) | .107 |
|  | Weight | 0.97 (0.93–1.01) | .183 |
|  | Tongue thickness | 0.84 (0.77–0.93) | < .001* |

Tongue echo intensity categories were defined using an exploratory cutoff value of 23.53 derived from receiver operating characteristic analysis.

OR, odds ratio; CI, confidence interval.

Logistic regression models were adjusted for age and sex.

An asterisk (*) indicates *P* < 0.05.

Model 1 represents the unadjusted baseline model including Yin-deficiency only and is shown for comparison with subsequent adjusted models.
