# Supplementary material for: Association between tongue ultrasonographic characteristics, Yin-deficiency constitution, and intrinsic capacity impairment in older adults: An exploratory cross-sectional study
Source: Medicine (Baltimore). 2026 Jul 10;105(28):e49571. doi: 10.1097/MD.0000000000049571 (PMC13363339; doi:10.1097/MD.0000000000049571)
Supplement: Supplementary file 3 [file medi-105-e49571-s003.docx]

**Table S3. Supplementary exploratory baseline characteristics and Integrated Care for Older People impairments according to the combination of receiver operating characteristic-derived high tongue echo intensity and Yin-deficiency constitution**

| **Variables** | **Reference group**  **(n = 95)** | **High tongue echo intensity and Yin-deficiency constitution**  **(n=28)** | ***P*-value** |
| --- | --- | --- | --- |
| **Age, years** | 71.8 ± 4.8 | 70.9 ± 3.5 | .304 |
| **Male** | 29 (30.5%) | 5 (17.9%) | .339 |
| **Height** | 157.6 ± 7.2 | 158.3 ± 6.8 | .617 |
| **Weight** | 61.0 ± 10.3 | 59.7 ± 7.3 | .448 |
| **Intrinsic capacity impairment** | | | |
| **Cognitive impairment** | 30 (31.6%) | 14 (50.0%) | .118 |
| **Limited mobility** | 28 (29.5%) | 12 (42.9%) | .272 |
| **Malnutrition** | 8 (8.4%) | 3 (10.7%) | 1.000 |
| **Visual impairment** | 33 (34.7%) | 18 (64.3%) | .010* |
| **Hearing loss** | 16 (16.8%) | 5 (17.9%) | 1.000 |
| **Depressive symptoms** | 13 (13.7%) | 7 (25.0%) | .256 |

Data are presented as mean ± standard deviation (SD) or n (%), as appropriate.

Continuous variables were compared using independent t tests.

Categorical variables were compared using chi-square tests.

An asterisk (*) indicates *P* < 0.05.

The reference group included participants without both high tongue echo intensity (>23.53) and Yin-deficiency constitution.
