# Supplementary material for: Association between tongue ultrasonographic characteristics, Yin-deficiency constitution, and intrinsic capacity impairment in older adults: An exploratory cross-sectional study
Source: Medicine (Baltimore). 2026 Jul 10;105(28):e49571. doi: 10.1097/MD.0000000000049571 (PMC13363339; doi:10.1097/MD.0000000000049571)
Supplement: Supplementary file 4 [file medi-105-e49571-s004.docx]

**Table S4. Supplementary exploratory multivariable logistic regression analyses of Integrated Care for Older People domain impairments associated with the combination of receiver operating characteristic-derived high tongue echo intensity and Yin-deficiency constitution**

| **Intrinsic capacity impairment** | **Adjusted OR (95% CI)** | ***P*-value** |
| --- | --- | --- |
| Cognitive impairment | 2.54 (1.04–6.22) | .041* |
| Limited mobility | 1.82 (0.76–4.38) | .183 |
| Malnutrition | 1.13 (0.27–4.65) | .868 |
| Visual impairment | 3.38 (1.39–8.24) | .007* |
| Hearing loss | 1.29 (0.41–4.03) | .666 |
| Depressive symptoms | 2.21 (0.77–6.40) | .143 |

OR, odds ratio; CI, confidence interval.

Separate multivariable logistic regression models were constructed for each Integrated Care for Older People domain.

All models were adjusted for age and sex.

An asterisk (*) indicates *P* < 0.05.
